# Gene expression profiling of macrophages: implications for an immunosuppressive effect of dissolucytotic gold ions

**DOI:** 10.1186/1476-9255-9-43

**Published:** 2012-11-09

**Authors:** Oliver Seifert, Andreas Matussek, Florence Sjögren, Robert Geffers, Chris D Anderson

**Affiliations:** 1 Division of Dermatology, Ryhov Hospital, S-55185, Jönköping, Sweden; 2 Department of Laboratory Medicine, Clinical Microbiology, Ryhov Hospital, Jönköping, Sweden; 3 Division of Dermatology, Department of Clinical and Experimental Medicine, Linköping University Hospital, Linköping, Sweden; 4 Genome Analytics, Helmholtz Centre for Infection Research, Braunschweig, Germany

**Keywords:** Gold, Macrophages, Inflammation, Rheumatoid arthritis

## Abstract

**Background:**

Gold salts has previously been used in the treatment of rheumatoid arthritis but have been replaced by biologicals such as TNF-α inhibitors. The mechanisms behind the anti-inflammatory effect of metallic gold ions are still unknown, however, recent data showed that charged gold atoms are released from pure metallic gold implants by macrophages via a dissolucytosis membrane, and that gold ions are taken up by local macrophages, mast cells and to some extent fibroblasts. These findings open the question of possible immunomodulatory effects of metallic gold and motivate efforts on a deeper understanding of the effect of metallic gold on key inflammatory cells as macrophages.

**Methods:**

Human macrophage cells (cell line THP-1) were grown on gold foils and intracellular uptake was analysed by autometallography. The impact of phagocytised gold ions on viability of THP-1 cells was investigated by trypan blue staining and TUNEL assay. The global gene expression profile of THP-1 cells after incorporation of gold ions was studied using microarray analysis comprising approximately 20,000 genes. The gene expression data was confirmed by measurement of secreted proteins.

**Results:**

Autometallography showed intracellular uptake of gold ions into THP-1 cells. No significant effect on viability of THP-1 cells was demonstrated. Our data revealed a unique gene expression signature of dissolucytotic THP-1 cells that had taken up gold ions. A large number of regulated genes were functionally related to immunomodulation. Gold ion uptake induced downregulation of genes involved in rheumatoid arthritis such as hepatocyte growth factor, tenascin-C, inhibitor of DNA binding 1 and 3 and matrix metalloproteinase 13.

**Conclusion:**

The data obtained in this study offer new insights into the mode of action of gold ions and suggest for the investigation of effects on other key cells and a possible future role of metallic gold as implants in rheumatoid arthritis or other inflammatory conditions.

## Background

The “noble” metal gold has been used in medicine over a long period, most recently in the treatment of rheumatoid arthritis (RA) [1-3]. Unpredictability of response to gold salts, occurrence of side effects and competition from modern effective but often expensive pharmaceuticals (e.g. biologics) have been problems hindering a broader therapeutic use of gold. Gold metal implants have also been used in veterinary medicine [4] and new immunological data from animal research provides deeper understanding of the potential therapeutic effects of gold [5-8]. Gold nanoparticles have been shown to have antiangiogenic properties [9] and use of gold nanoparticles in new oncology methods [10] is another area of recent development.

Despite the use of gold in both human and veterinary medicine the possible mechanism of action of gold is yet not fully understood. Gold ions have been shown to inhibit the lysosomal enzymes of phagocytotic cells [11], to decrease the number of macrophages in the synovial membrane [7] and to reduce production of pro-inflammatory cytokines in cell culture [12]. Danscher was the first to show that charged gold atoms are released from pure metallic gold implants [13]. He and his group demonstrated that macrophages home in on the bio-membrane that covers metallic gold implants. Gold ions are released from the metallic gold surface into the ‘dissolution membrane’ under the sway of the adhering macrophages [14].

Synovial macrophages are key players in RA [15]. They are involved in the initiation and perpetuation of inflammation, leukocyte adhesion and migration, matrix degradation and angiogenesis. Macrophages express adhesion molecules, chemokine receptors and other surface antigens and secrete a number of chemokines, cytokines, growth factors, proteases and other mediators. Macrophages and their products are key players in the pathogenesis of RA and other inflammatory diseases and may be promising therapeutic targets [16].

Autometallography (AMG) has enabled the observation of movement of gold ions from the dissolution membrane to the intercellular environment from which they enter macrophages, mast cells and other cells in the local environment [13]. Presence of gold both in the intercellular space and within cells has been demonstrated in animal studies [13,17]. It has also been noted that ionisation occurs more rapidly in inflamed tissue than in normal tissue, and that the greater the surface area the greater the ionisation of gold [18].

The aim of the present paper was to study whether the human macrophage cell-line THP-1, which shares many properties with human monocyte-derived macrophages, grown on gold foils of 24-carat gold could lead to liberation and cellular incorporation of gold ions and whether this process was associated with changes in gene expression and subsequent protein secretion and cell viability.

## Methods

### Cell culture

The human macrophage cell line THP-1 (European Collection of Cell Cultures, ECACC) shares many properties with human monocyte-derived macrophages. THP-1 cells were maintained under standard culture conditions as previously described [19-21]. Briefly, cells were grown in HEPES-buffered RPMI 1640 (GIBCO, Invitrogen, Paisley, UK) supplemented with 10% fetal calf serum (PAA, Pasching, Austria), 2 mM L-glutamine (Invitrogen), 100 U/ml penicillin, 1% non-essential amino acids and 0.1 mg/ml streptomycin (Sigma-Aldrich, Deisenhofen, Germany). Cell cultures were maintained at 37°C in a humidified incubator with 5% carbon dioxide. Adherent cells were loosened using a small plastic scraper.

THP-1 cells growing on gold foils (1 mm thick, 99.95 pure gold, size 25 × 25mm, Alfa Aesar, Karlsruhe, Germany) were placed in Petri dishes, 4 cm diameter. 3 ml medium containing 5 × 10^6^/ml cells were added. Cells were harvested after 1, 2, 3 and 4 days. Cells grown directly on the bottom of the standard Petri dishes served as control cells.

### Cells preparing for AMG gold tracing

Prior to AMG gold tracing, the cells growing on gold foils were mechanically loosened in 5 ml cell medium. The cell pellet was resuspended and dispersed onto microscopic slides at 500 g for 3 min by a cytocentrifuge (Cytospin 2, Shandon, Axel Johnson Labsystem, Stockholm, Sweden). The slides were then placed for 1 hour under an UVA lamp (TL20W/09 N with a wave length range 320-400 nm, Philips, Stockholm, Sweden) [13]. Following this reduction of gold by ionization, the slides were then silver enhanced for 1 hour by AMG [17], a process that deposits silver on gold nanoparticles. After 10 minutes in the developer the slides were washed with water and counterstained with 0,5% toluidine blue at pH 4 for 10 min. Following washing the slides were dehydrated in alcohol, mounted and coverslipped.

### DNA microarray hybridization and analysis

RNA from three different experiments was pooled and the mean of two different experiments was calculated. Double quality and integrity of the total RNA was controlled on an Agilent Technologies 2100 Bioanalyzer (Agilent Technologies, Waldbronn, Germany). 500 ng of total RNA were applied for Cy3-labelling reaction using the one color Quick Amp Labeling protocol (Agilent Technologies). Labeled cRNA was hybridized to Agilent’s human 4 × 44k microarrays for 16 hours at 68°C and scanned using the Agilent DNA Microarray Scanner. Expression values were calculated by the software package Feature Extraction 10.5.1.1 (Agilent Technologies). Statistical analysis of the expression data was performed using the Gene Spring Software package (Agilent Technologies). Genes significantly expressed (ANOVA, p < 0.05) and with a fold change ≥ 2 were considered differentially regulated.

### Enzyme-linked immunosorbent assay (ELISA)

Early growth response 1 (EGR1), fatty acid desaturase 1 (FADS1) and lymphotoxin B (LTB) were measured in cell culture media from THP-1 cells grown on gold foils and control cells using specific ELISA’s according to the manufacturer’s instructions (Antibodies-online, Aachen, Germany). Cell culture supernatants from three independent experiments were analysed.

### Determination of cell viability

To analyse the viability of THP-1 cells trypan blue staining blue (Sigma–Aldrich) was performed and to confirm these results the TUNEL assay (ApopTag® Plus Peroxidase *In Situ* Apoptosis Detection Kit, Millipore, Billerica, USA) was performed, according to the manufacturer’s instructions. TUNEL-positive cells are presented as percentage of 100 cells.

### Data analysis

Each experiment was repeated three times. Results are expressed as means ± standard deviation. Differences among groups were analyzed using the students *t*-test and p values < 0.05 were considered significant.

#### Online supplemental material

A complete table of the 1028 differentially regulated genes (FC > 2) is provided as supplementary material (Additional file 1: Table S1). The entire microarray dataset is available at GEO database under Acc. GSE37814 ( http://www.ncbi.nlm.nih.gov/projects/geo/).

## Results

### Gold ions are incorporated by THP-1 cells

**Figure 1:**
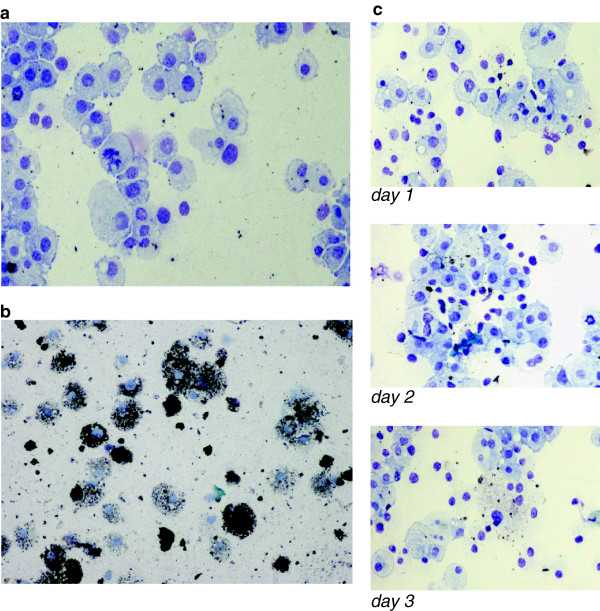
**a: Autometallographic analysis of THP-1 cells cultured without gold contact after 4 days.b:** Autometallographic analysis of THP-1 cells grown on gold foils after 4 days. **c:** Autometallographic analysis of THP-1 cells grown on gold foils after 1, 2 and 3 days.

Visualisation of metallic gold incorporated by THP-1 cells by AMG tracing showed increased uptake of gold ions (dissolucytosis) in the cultured cells after 4 days growth on gold foils (Figure 1a,b). No significant uptake of gold was seen in cells cultured on gold foils for 1, 2 or 3 days (Figure 1c)

### Cell viability is not influenced by gold ions

**Figure 2:**
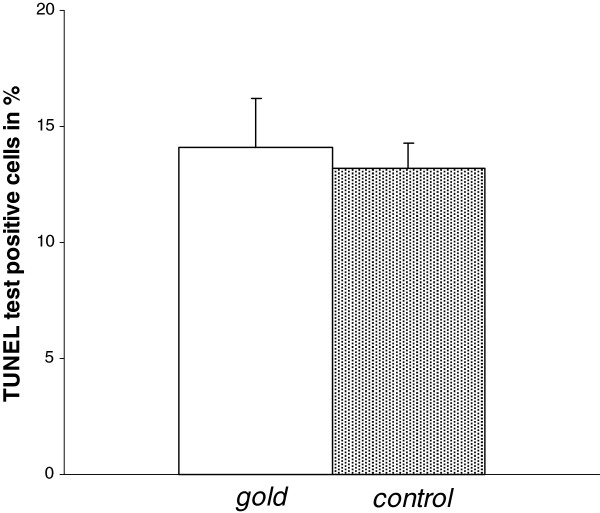
No significant difference in amount of TUNEL test positive THP-1 cells was seen between cells grown on gold foils and control cells in percent after 4 days (n = 3, p > 0.05).

Cell viability was >95% as determined by trypan-exclusion in both cell groups. Our results showed that 14.1% of THP-1 cells were TUNEL test positive in the gold group and 13.2% in the control group, respectively (Figure 2). There was no significant difference in both groups (n = 3, p > 0.05).

### Dissolucytosis of gold ions effect protein secretion

**Figure 3:**
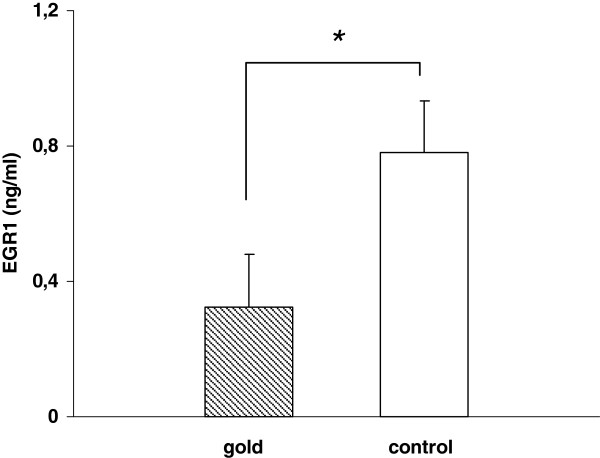
THP-1 cells grown on gold foils showed significant decreased expression of early growth response 1 (EGR1) protein in cell culture supernatants compared with control cells after 4 days (n = 3, p < 0.05).

**Figure 4:**
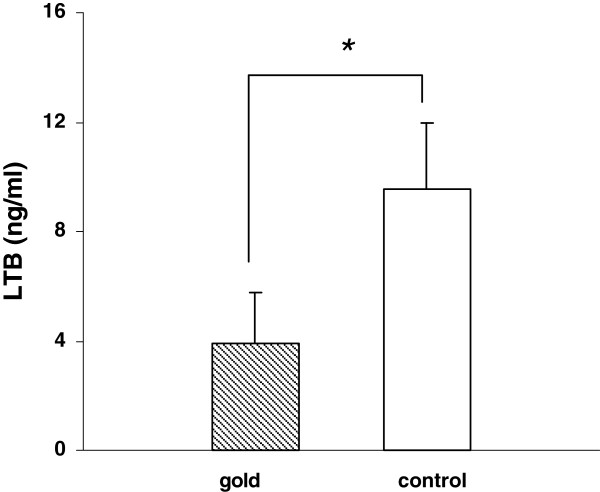
THP-1 cells grown on gold foils showed significant decreased expression of lymphotoxin B (LTB) protein in cell culture supernatants compared with control cells after 4 days (n = 3, p < 0.05).

**Figure 5:**
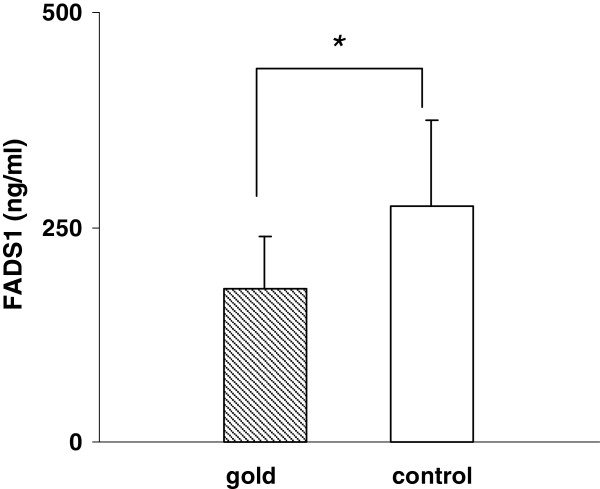
THP-1 cells grown on gold foils showed significant decreased expression of fatty acid desaturase 1 (FADS1) protein in cell culture supernatants compared with control cells after 4 days (n = 3, p < 0.05).

To determine the effect of gold phagocytosis on the production of three selected secreted proteins specific ELISA’s of cell culture supernatants were performed. Our results showed significant decreased expression of EGR1, LTB and FADS1 (Figure 3, 4, 5) after 4 days in cells that incorporated gold compared to control cells.

### Genes involved in rheumatoid arthritis and inflammation are regulated by dissolucytosis of gold ions

Analysis of the 20 000 genes investigated by DNA microarray revealed a total of 1028 (fold change (FC) ≥ 2) respectively 156 (FC ≥ 3) differently regulated genes in THP-1 cells cultured on gold foils compared to control cells without gold contact. Of these 1028 genes 462 were downregulated and 566 upregulated. In the gene group with a FC ≥ 3 54 genes were upregulated and 102 genes were downregulated. A complete list of these regulated genes is provided as supplementary material (Additional file 1: Table S1). Among these genes hepatocyte growth factor (HGF), tenascin-C (TNC), inhibitor of DNA binding 1 and 3 (ID1 and 3) and matrix metalloproteinase 13 (MMP13) were of special interest since they were downregulated by gold dissolucytosis and they are directly involved in RA pathogenesis.

To study the effect of incorporated gold ions on inflammation a subanalysis of a cluster of genes involved in inflammation was performed and revealed differential regulation of 34 genes (FC ≥ 2, 16 downregulated and 18 upregulated genes, Table 1). A major group of genes encode for proteins with immune regulatory functions as LTB, EGR1, FADS1 and other genes are substantially involved in RA as fas-ligand (FASLG) and CLC5A.

**Table 1:** Selected genes involved in inflammation differentially regulated (FC ≥ 2) in THP-1 cells after dissolucytosis of gold compared to control THP-1 cells

**Fold change**	**Regulation**	**GeneSymbol**	**EntrezGene**
2,1	down	CLEC5A	23601
2,7	up	LAT	27040
2,0	down	VISA	57506
2,0	up	LTBR	4055
4,2	down	MYST3	7994
2,7	down	CLEC4M	10332
2,3	down	IRF7	3665
3,0	Down	FADS1	3992
2,1	up	IFI27	3429
3,3	up	CXCL12	6387
2,2	up	CD86	942
2,5	down	CCL26	10344
2,1	up	OLR1	4973
2,5	up	MS4A1	931
2,1	up	CLEC7A	64581
2,1	up	CTSE	1510
2,0	down	IGHD	3495
3,7	up	INHBA	3624
2,2	up	TCF12	6938
4,4	down	EGR1	1958
2,9	up	CPLX2	10814
2,0	down	FEZ2	9637
3,2	up	FASLG	356
3,3	down	LTB	4050
2,5	up	HLA-DOA	3111
2,1	down	ETS1	2113
2,5	up	CTSS	1520
2,7	up	IL1F8	27177
2,2	down	IGSF6	10261
2,6	up	HLA-C	3107
2,6	down	C1QB	713
2,9	up	CCRL1	51554
4,9	down	BCL11A	53335
2,5	down	DOCK2	1794

## Discussion

Presently, the use of gold salts in the treatment of RA is of little clinical interest in western countries due to the common occurrence of side effects and relatively low and unpredictable efficacy [22]. However, gold ions, dissolucytotically released from metallic gold implants, have been shown to be anti-inflammatory [18,23-26]. Together with recent findings of decreased articular pain and inflammation in veterinary use [6,8], it is of interest to analyse the mode of action of metallic gold which can be applied to specific points of inflammation without systemic effects.

The aetiology of RA remains unknown, however, many cell types such as macrophages, lymphocytes and fibroblasts, and cytokines have been implicated in the pathogenesis of RA [27]. A key role for macrophages has been suggested, in part by successful treatment with blockade of TNF-α. In inflamed tissue TNF-α production is widely performed by activated macrophages [28]. In RA, the number of macrophages in the synovial tissue correlates to the degree of joint erosion [29], and increased number of macrophages are an early hallmark of active disease [30]. Some mechanisms have been put forward to explain the efficacy of gold salts in RA, including direct effects on synovial macrophages since gold salts are predominantly uptaken by macrophages [31]. However, the exact mode of action of gold in RA and other conditions having pathogenetic similarities is still unclear.

A limitation in the present study is the sole use of the human THP-1 cell line which shares many properties with human monocyte-derived macrophages [32] but does not resemble monocytes-macrophages isolated from human donors. This has been taken into account interpreting the results in this study. Our results showed that THP-1 cells incorporate gold ions bio-released from metallic gold. Gold uptake had no effect on the viability of THP-1 cells indicating that anti-inflammatory effects might not be mediated via macrophage cell death although apoptotic processes cannot totally be excluded since only one apoptotic test was performed in this study. However, we found that uptake of gold modulated the gene expression profile in THP-1 cells, differentially regulating a large number of genes (1028 genes with a FC ≥ 2 and 156 genes with a FC ≥ 3).

Since we were most interested in the effect of gold on inflammation we analysed a cluster of genes involved in inflammation and found 34 genes differentially regulated. In this group we found decreased expression of the LTB gene and decreased protein secretion in cell culture supernatants of THP-1 cells after gold uptake. LTB is involved in chronic inflammation and autoimmunity and LTB antibodies have been tested for inhibiting LTB mediated inflammation [33]. O’Rourke et al. found high levels of LTB gene expression in RA synovium and showed a significant positive correlation between LTB synovial gene expression and pain VAS score. They conclude that LTB may play a role in RA disease pathogenesis by contributing to a more intense inflammatory reaction in the synovium [34]. Both EGR1 gene and protein expression was downregulated in our experiments. Several studies demonstrated the significant role of EGR1 in inflammation [35]. EGR1 protein is expressed in T-cells and is involved in the acute phase of the IL-4 transcription in response to T-cell receptor stimulation [36]. Interestingly, EGR1 is directly involved in TNF-α mediated upregulation of prostaglandin E2 leading to inflammation and arthritis [37]. These results suggest a possible role for gold in the treatment of RA by suppressing expression of LTB and EGR1.

FADS1 gene encodes for delta-5 desaturase, a key enzyme in polyunsaturated fatty acid metabolism catalyzing the production of pro-inflammatory arachidonic acid (AA) and eicosanoids, which are biologically active at very low concentrations [38]. Gold exposure of THP-1 cells revealed decreased expression of FADS1 gene and decreased protein secretion. Future studies have to clear a functional role of gold induced suppression of FADS1 gene in inhibiting inflammation.

Our inflammation gene cluster showed decreased CLEC5A expression in THP-1 cells after gold uptake. CLEC5A is a key regulator of synovial injury and bone erosion during autoimmune joint inflammation. Activation of CLEC5A leads to enhanced recruitment of inflammatory macrophages and neutrophils to the joint and promotes bone erosion. Functional blockade of CLEC5A reduces the clinical signs of autoimmune joint inflammation. These findings suggest that CLEC5A may be a therapeutic target for treatment of immune-mediated skeletal disorders [39].

Surgical synovectomy to remove the inflammatory synovium can temporarily ameliorate rheumatoid inflammation and delay the progress of joint destruction. An efficient medically induced programmed cell death (apoptosis) in the rheumatoid synovium might play a role similar to synovectomy but without surgical tissue damage. Gene transfer of FASLG has increased the frequency of apoptotic cells in mouse and rabbit arthritic synovium. A previous study showed that repeated FASLG gene transfer could remove human inflammatory synovial tissue *in situ* and function as a ‘gene scalpel’ for molecular synovectomy to arrest inflammatory synovium at an early stage of RA [40]. The results obtained in our study showed that gold uptake induced FASLG gene expression in macrophages.

Interestingly, among the genes strongly regulated with a FC ≥ 3 after dissolucytosis of gold ions several are directly involved in the pathogenesis of RA.

HGF has been shown to inhibit osteoblast differentiation and plasma levels of HGF predict joint damage in RA suggesting that HGF plays a role in RA joint destruction [41]. Other studies are linking HGF to angiogenesis in RA [42] and HGF is highly upregulated in synovial fluids of patients with RA [43]. Our data revealed strong downregulation of HGF after gold ion uptake implicating a potential new anti-inflammatory pathway of gold.

Expression of inhibitor of differentiation (ID) gene family is considered to be relevant to the pathogenesis of RA, because ID family genes have been shown to play a role in cell proliferation and angiogenesis and it was proposed that inhibition of expression and/or function of ID1 and 3 may potentially be of therapeutic value for conditions associated with pathological angiogenesis [44]. A previous study showed increased mRNA and immunohistochemistry staining of ID1 and 3 in the synovium of RA patients [45] and interestingly, our data showed strong downregulation of ID1 and 3 by gold ion uptake implicating a mode of action for gold.

In a recent publication Midwood et al. revealed TNC as a novel endogenous activator of TLR4-mediated immunity that mediates persistent synovial inflammation and tissue destruction in arthritic joint disease [46]. Our array results showed strong downregulation of TNC as a result of gold ion uptake. Numerous recent studies highlight the important role of TNC in RA [47-49] supporting our hypothesis that a potential anti-inflammatory effect gold is mediated by suppressing TNC production.

Matrix metalloproteinases are known to contribute to the development of RA [50]. MMP13 has been shown to be associated with synovitis in RA [51] and recently, studies of leflunomide and tacrolimus, two active substances in the treatment of RA, were found to be partly active by suppressing expression of MMP13 [52,53]. This is in line with our findings showing suppression of MMP13 expression by gold.

The anti-TNF-α antibody adalimumab is used in the treatment of RA and beside blocking TNF-α Adalimumab increases CD36 on human macrophages [54]. Our data revealed upregulation of CD36 in macrophages after gold uptake suggesting a possible anti-inflammatory effect of gold via CD36 upregulation.

The nuclear hormone receptors NR4A1 has been implicated in RA and apoptosis. The purine antimetabolite 6-Mercaptopurine (6-MP), which is widely used as an anti-neoplastic and anti-inflammatory drug, induces NR4A1 expression [55]. DeSilva et al. showed that NR4A1 overexpression in T cells attenuates the development and progression of collagen-induced arthritis, CIA, probably by promoting activation-induced T cell apoptosis and by inhibiting collagen type II specific antibody production [56]. Further studies are needed to show whether and how gold induced expression of NR4A1 in macrophages is involved in the anti-inflammatory effect of gold in RA.

Beside macrophages T- and B-lymphocytes play an important role in RA [57] and it is of interest to further study the effect of gold ions on the gene expression profile of these cells. A limitation for such studies is that contact hypersensitivity to gold is common. The lymphocyte transformation test (LTT) has been studied in the diagnosis of contact hypersensitivity to gold showing significantly higher stimulation indexes for LTT [58,59] in patients sensitized to gold. Therefore patch testing should be performed in future studies on gold including lymphocytes from human donors.

## Conclusions

The present study revealed a unique gene expression profile of THP-1 cells after uptake of dissolucytotic gold ions indicating possible mechanisms for the anti-inflammatory effect of gold. Further studies are needed to show whether metallic gold might be useful in safe local treatment of inflammatory diseases which would not be associated with the toxicity encountered with systemic gold administration.

## Competing interests

The authors declare that they have no competing interests.

## Authors’ contributions

OS and CA made substantial contributions to conception, design, analysis and interpretation of data and have been involved in drafting the manuscript and revising it critically for important intellectual content. OS carried out the ELISA and TUNEL assay and performed the statistical analysis. AM and RG carried out the microarray analysis, performed statistical analysis and have been involved in drafting the manuscript and revising it critically for important intellectual content. FS carried out autometallography and drafted the manuscript. All authors read the final manuscript and have given final approval of the version to be published.

## Supplementary Material

Additional file 1 **Table S1.** Selected genes differentially regulated (FC ≥ 3) in THP-1 cells after dissolucytosis of gold compared to control THP-1 cells. Click here for file

## References

[B1] Burmester GR Molecular mechanisms of action of gold in treatment of rheumatoid arthritis--an update Z Rheumatol 2001 60 167 173 10.1007/s003930170065 11475604

[B2] Simon LS Yocum D New and future drug therapies for rheumatoid arthritis Rheumatol (Oxford) 2000 39 Suppl 1 36 42 10.1093/oxfordjournals.rheumatology.a031493 11001378

[B3] Simon LS DMARDs in the treatment of rheumatoid arthritis: current agents and future developments Int J Clin Pract 2000 54 243 249 10912314

[B4] Jaeger GT Larsen S Soli N Moe L Two years follow-up study of the pain-relieving effect of gold bead implantation in dogs with hip-joint arthritis Acta Vet Scand 2007 49 9 10.1186/1751-0147-49-9 17381835 PMC1851017

[B5] Canumalla AJ Al-Zamil N Phillips M Isab AA Shaw CF 3rd Redox and ligand exchange reactions of potential gold(I) and gold(III)-cyanide metabolites under biomimetic conditions J Inorg Biochem 2001 85 67 76 10.1016/S0162-0134(00)00224-5 11377697

[B6] Bondeson J Sundler R Auranofin inhibits the induction of interleukin 1 beta and tumor necrosis factor alpha mRNA in macrophages Biochem Pharmacol 1995 50 1753 1759 10.1016/0006-2952(95)02030-6 8615853

[B7] Yanni G Nabil M Farahat MR Poston RN Panayi GS Intramuscular gold decreases cytokine expression and macrophage numbers in the rheumatoid synovial membrane Ann Rheum Dis 1994 53 315 322 10.1136/ard.53.5.315 8017985 PMC1005330

[B8] Jaeger GT Larsen S Soli N Moe L Double-blind, placebo-controlled trial of the pain-relieving effects of the implantation of gold beads into dogs with hip dysplasia Vet Rec 2006 158 722 726 10.1136/vr.158.21.722 16731702

[B9] Mukherjee P Bhattacharya R Wang P Wang L Basu S Nagy JA Atala A Mukhopadhyay D Soker S Antiangiogenic properties of gold nanoparticles Clin Cancer Res 2005 11 3530 3534 10.1158/1078-0432.CCR-04-2482 15867256

[B10] Jin H Hong B Kakar SS Kang KA Tumor-specific nano-entities for optical detection and hyperthermic treatment of breast cancer Adv Exp Med Biol 2008 614 275 284 10.1007/978-0-387-74911-2_31 18290338

[B11] Persellin RH Ziff M The effect of gold salt on lysosomal enzymes of the peritoneal macrophage Arthritis Rheum 1966 9 57 65 10.1002/art.1780090107 5006477

[B12] Traber KE Okamoto H Kurono C Baba M Saliou C Soji T Packer L Okamoto T Anti-rheumatic compound aurothioglucose inhibits tumor necrosis factor-alpha-induced HIV-1 replication in latently infected OM10.1 and Ach2 cells Int Immunol 1999 11 143 150 10.1093/intimm/11.2.143 10069412

[B13] Danscher G In vivo liberation of gold ions from gold implants. Autometallographic tracing of gold in cells adjacent to metallic gold Histochem Cell Biol 2002 117 447 452 10.1007/s00418-002-0400-8 12029492

[B14] Larsen A Stoltenberg M Danscher G In vitro liberation of charged gold atoms: autometallographic tracing of gold ions released by macrophages grown on metallic gold surfaces Histochem Cell Biol 2007 128 1 6 10.1007/s00418-007-0295-5 17549510

[B15] Szekanecz Z Koch AE Macrophages and their products in rheumatoid arthritis Curr Opin Rheumatol 2007 19 289 295 10.1097/BOR.0b013e32805e87ae 17414958

[B16] Okamoto H Hoshi D Kiire A Yamanaka H Kamatani N Molecular targets of rheumatoid arthritis Inflamm Allergy Drug Targets 2008 7 53 66 10.2174/187152808784165199 18473901

[B17] Danscher G Stoltenberg M Silver enhancement of quantum dots resulting from (1) metabolism of toxic metals in animals and humans, (2) in vivo, in vitro and immersion created zinc-sulphur/zinc-selenium nanocrystals, (3) metal ions liberated from metal implants and particles Prog Histochem Cytochem 2006 41 57 139 10.1016/j.proghi.2006.06.001 16949439

[B18] Larsen A Kolind K Pedersen DS Doering P Pedersen MO Danscher G Penkowa M Stoltenberg M Gold ions bio-released from metallic gold particles reduce inflammation and apoptosis and increase the regenerative responses in focal brain injury Histochem Cell Biol 2008 130 681 692 10.1007/s00418-008-0448-1 18542984

[B19] Zdolsek JM Olsson GM Brunk UT Photooxidative damage to lysosomes of cultured macrophages by acridine orange Photochem Photobiol 1990 51 67 76 2304980 10.1111/j.1751-1097.1990.tb01685.x

[B20] Brunk UT Dalen H Roberg K Hellquist HB Photo-oxidative disruption of lysosomal membranes causes apoptosis of cultured human fibroblasts Free Radic Biol Med 1997 23 616 626 10.1016/S0891-5849(97)00007-5 9215807

[B21] Li W Yuan X Nordgren G Dalen H Dubowchik GM Firestone RA Brunk UT Induction of cell death by the lysosomotropic detergent MSDH FEBS Lett 2000 470 35 39 10.1016/S0014-5793(00)01286-2 10722841

[B22] Helliwell PS Taylor WJ Treatment of psoriatic arthritis and rheumatoid arthritis with disease modifying drugs – comparison of drugs and adverse reactions J Rheumatol 2008 35 472 476 18203324

[B23] Danscher G Larsen A Effects of dissolucytotic gold ions on recovering brain lesions Histochem Cell Biol 2010 133 367 373 10.1007/s00418-010-0681-2 20237795

[B24] Pedersen MO Larsen A Stoltenberg M Penkowa M Bio-released gold ions modulate expression of neuroprotective and hematopoietic factors after brain injury Brain Res 2010 1307 1 13 19840777 10.1016/j.brainres.2009.10.028

[B25] Pedersen MO Larsen A Pedersen DS Stoltenberg M Penkowa M Metallic gold reduces TNFalpha expression, oxidative DNA damage and pro-apoptotic signals after experimental brain injury Brain Res 2009 1271 103 113 19328189 10.1016/j.brainres.2009.03.022

[B26] Pedersen MO Larsen A Pedersen DS Stoltenberg M Penkova M Metallic gold treatment reduces proliferation of inflammatory cells, increases expression of VEGF and FGF, and stimulates cell proliferation in the subventricular zone following experimental traumatic brain injury Histol Histopathol 2009 24 573 586 19283666 10.14670/HH-24.573

[B27] Kinne RW Stuhlmuller B Burmester GR Cells of the synovium in rheumatoid arthritis. Macrophages Arthritis Res Ther 2007 9 224 10.1186/ar2333 18177511 PMC2246244

[B28] Tracey D Klareskog L Sasso EH Salfeld JG Tak PP Tumor necrosis factor antagonist mechanisms of action: a comprehensive review Pharmacol Ther 2008 117 244 279 10.1016/j.pharmthera.2007.10.001 18155297

[B29] Mulherin D Fitzgerald O Bresnihan B Synovial tissue macrophage populations and articular damage in rheumatoid arthritis Arthritis Rheum 1996 39 115 124 10.1002/art.1780390116 8546720

[B30] Singh JA Pando JA Tomaszewski J Schumacher HR Quantitative analysis of immunohistologic features of very early rheumatoid synovitis in disease modifying antirheumatic drug- and corticosteroid-naive patients J Rheumatol 2004 31 1281 1285 15229944

[B31] Vernon-Roberts B Dore JL Jessop JD Henderson WJ Selective concentration and localization of gold in macrophages of synovial and other tissues during and after chrysotherapy in rheumatoid patients Ann Rheum Dis 1976 35 477 486 10.1136/ard.35.6.477 1087551 PMC1006590

[B32] Zetterstrom CK Jiang W Wahamaa H Ostberg T Aveberger AC Schierbeck H Lotze MT Andersson U Pisetsky DS Erlandsson Harris H Pivotal advance: inhibition of HMGB1 nuclear translocation as a mechanism for the anti-rheumatic effects of gold sodium thiomalate J Leukoc Biol 2008 83 31 38 17913975 10.1189/jlb.0507323

[B33] Remouchamps C Boutaffala L Ganeff C Dejardin E Biology and signal transduction pathways of the Lymphotoxin-alphabeta/LTbetaR system Cytokine Growth Factor Rev 2011 22 301 310 10.1016/j.cytogfr.2011.11.007 22152226

[B34] O’Rourke KP O’Donoghue G Adams C Mulcahy H Molloy C Silke C Molloy M Shanahan F O’Gara F High levels of Lymphotoxin-Beta (LT-Beta) gene expression in rheumatoid arthritis synovium: clinical and cytokine correlations Rheumatol Int 2008 28 979 986 10.1007/s00296-008-0574-z 18379788

[B35] Tureyen K Brooks N Bowen K Svaren J Vemuganti R Transcription factor early growth response-1 induction mediates inflammatory gene expression and brain damage following transient focal ischemia J Neurochem 2008 105 1313 1324 10.1111/j.1471-4159.2008.05233.x 18208539 PMC2603292

[B36] Lohoff M Giaisi M Kohler R Casper B Krammer PH Li-Weber M Early growth response protein-1 (Egr-1) is preferentially expressed in T helper type 2 (Th2) cells and is involved in acute transcription of the Th2 cytokine interleukin-4 J Biol Chem 2010 285 1643 1652 10.1074/jbc.M109.011585 19915002 PMC2804322

[B37] Fahmi H mPGES-1 as a novel target for arthritis Curr Opin Rheumatol 2004 16 623 627 10.1097/01.bor.0000129664.81052.8e 15314505

[B38] Martinelli N Girelli D Malerba G Guarini P Illig T Trabetti E Sandri M Friso S Pizzolo F Schaeffer L FADS genotypes and desaturase activity estimated by the ratio of arachidonic acid to linoleic acid are associated with inflammation and coronary artery disease Am J Clin Nutr 2008 88 941 949 18842780 10.1093/ajcn/88.4.941

[B39] Joyce-Shaikh B Bigler ME Chao CC Murphy EE Blumenschein WM Adamopoulos IE Heyworth PG Antonenko S Bowman EP McClanahan TK Myeloid DAP12-associating lectin (MDL)-1 regulates synovial inflammation and bone erosion associated with autoimmune arthritis J Exp Med 2010 207 579 589 10.1084/jem.20090516 20212065 PMC2839155

[B40] Zhang H Gao G Clayburne G Schumacher HR Elimination of rheumatoid synovium in situ using a Fas ligand ‘gene scalpel’ Arthritis Res Ther 2005 7 R1235 R1243 10.1186/ar1811 16277676 PMC1297566

[B41] Grandaunet B Syversen SW Hoff M Sundan A Haugeberg G van Der Heijde D Kvien TK Standal T Association between high plasma levels of hepatocyte growth factor and progression of radiographic damage in the joints of patients with rheumatoid arthritis Arthritis Rheum 2011 63 662 669 10.1002/art.30163 21360495

[B42] Maruotti N Cantatore FP Crivellato E Vacca A Ribatti D Angiogenesis in rheumatoid arthritis Histol Histopathol 2006 21 557 566 16493585 10.14670/HH-21.557

[B43] Yukioka K Inaba M Furumitsu Y Yukioka M Nishino T Goto H Nishizawa Y Morii H Levels of hepatocyte growth factor in synovial fluid and serum of patients with rheumatoid arthritis and release of hepatocyte growth factor by rheumatoid synovial fluid cells J Rheumatol 1994 21 2184 2189 7699616

[B44] Sakurai D Tsuchiya N Yamaguchi A Okaji Y Tsuno NH Kobata T Takahashi K Tokunaga K Crucial role of inhibitor of DNA binding/differentiation in the vascular endothelial growth factor-induced activation and angiogenic processes of human endothelial cells J Immunol 2004 173 5801 5809 15494533 10.4049/jimmunol.173.9.5801

[B45] Sakurai D Yamaguchi A Tsuchiya N Yamamoto K Tokunaga K Expression of ID family genes in the synovia from patients with rheumatoid arthritis Biochem Biophys Res Commun 2001 284 436 442 10.1006/bbrc.2001.4974 11394898

[B46] Midwood K Sacre S Piccinini AM Inglis J Trebaul A Chan E Drexler S Sofat N Kashiwagi M Orend G Tenascin-C is an endogenous activator of Toll-like receptor 4 that is essential for maintaining inflammation in arthritic joint disease Nat Med 2009 15 774 780 10.1038/nm.1987 19561617

[B47] Ruhmann M Piccinini AM Kong PL Midwood KS Endogenous activation of adaptive immunity: Tenascin-C drives IL-17 synthesis in arthritic joint disease Arthritis Rheum 2012 64 7 2179 2190 10.1002/art.34401 22275298

[B48] Sofat N Robertson SD Hermansson M Jones J Mitchell P Wait R Tenascin-C fragments are endogenous inducers of cartilage matrix degradation Rheumatol Int 2011 32 9 2809 2817 21874326 10.1007/s00296-011-2067-8 PMC3429773

[B49] Goh FG Piccinini AM Krausgruber T Udalova IA Midwood KS Transcriptional regulation of the endogenous danger signal tenascin-C: a novel autocrine loop in inflammation J Immunol 2010 184 2655 2662 10.4049/jimmunol.0903359 20107185

[B50] Burrage PS Mix KS Brinckerhoff CE Matrix metalloproteinases: role in arthritis Front Biosci 2006 11 529 543 10.2741/1817 16146751

[B51] Wernicke D Seyfert C Gromnica-Ihle E Stiehl P The expression of collagenase 3 (MMP-13) mRNA in the synovial tissue is associated with histopathologic type II synovitis in rheumatoid arthritis Autoimmunity 2006 39 307 313 10.1080/08916930600807709 16891219

[B52] Migita K Miyashita T Ishibashi H Maeda Y Nakamura M Yatsuhashi H Ida H Kawakami A Aoyagi T Kawabe Y Eguchi K Suppressive effect of leflunomide metabolite (A77 1726) on metalloproteinase production in IL-1beta stimulated rheumatoid synovial fibroblasts Clin Exp Immunol 2004 137 612 616 10.1111/j.1365-2249.2004.02555.x 15320915 PMC1809130

[B53] Migita K Miyashita T Maeda Y Aoyagi T Kawabe Y Nakamura M Yatsuhashi H Ishibashi H Eguchi K FK506 suppresses the stimulation of matrix metalloproteinase 13 synthesis by interleukin-1beta in rheumatoid synovial fibroblasts Immunol Lett 2005 98 194 199 10.1016/j.imlet.2004.11.014 15860218

[B54] Boyer JF Balard P Authier H Faucon B Bernad J Mazieres B Davignon JL Cantagrel A Pipy B Constantin A Tumor necrosis factor alpha and adalimumab differentially regulate CD36 expression in human monocytes Arthritis Res Ther 2007 9 R22 10.1186/ar2133 17335569 PMC1906797

[B55] Wansa KD Muscat GE TRAP220 is modulated by the antineoplastic agent 6-Mercaptopurine, and mediates the activation of the NR4A subgroup of nuclear receptors J Mol Endocrinol 2005 34 835 848 10.1677/jme.1.01739 15956351

[B56] De Silva S Han S Zhang X Huston DP Winoto A Zheng B Reduction of the incidence and severity of collagen-induced arthritis by constitutive Nur77 expression in the T cell lineage Arthritis Rheum 2005 52 333 338 10.1002/art.20736 15641076

[B57] Choy E Understanding the dynamics: pathways involved in the pathogenesis of rheumatoid arthritis Rheumatol (Oxford) 2012 51 Suppl 5 v3 11 10.1093/rheumatology/kes113 22718924

[B58] Vamnes JS Gjerdet NR Morken T Moe G Matre R In vitro lymphocyte reactivity to gold compounds in the diagnosis of contact hypersensitivity Contact Dermatitis 1999 41 156 160 10.1111/j.1600-0536.1999.tb06108.x 10475515

[B59] Christiansen J Farm G Eid-Forest R Anderson C Cederbrant K Hultman P Interferon-gamma secreted from peripheral blood mononuclear cells as a possible diagnostic marker for allergic contact dermatitis to gold Contact Dermatitis 2006 55 101 112 10.1111/j.1600-0536.2006.00908.x 16930235

