# Supplementary material for: Gene expression profiling of macrophages: implications for an immunosuppressive effect of dissolucytotic gold ions
Source: J Inflamm (Lond). 2012 Nov 9;9:43. doi: 10.1186/1476-9255-9-43 (PMC3526405; doi:10.1186/1476-9255-9-43)
Supplement: Additional file 1 — Table S1. Selected genes differentially regulated (FC ≥ 3) in THP-1 cells after dissolucytosis of gold compared to control THP-1 cells. [file 1476-9255-9-43-S1.doc]

**Table S1** Selected genes differentially regulated (FC ≥ 3) in THP-1 cells after dissolucytosis of gold compared to control THP-1 cells.

| **Fold change** | **Regulation** | **GeneSymbol** | **EntrezGene** |
| --- | --- | --- | --- |
|  |  |  |  |
| 3,3 | down | THC2729213 |  |
| 3,7 | down | CCDC94 | 55702 |
| 3,2 | down | A_24_P144625 |  |
| 3,0 | down | PIK4CB | 5298 |
| 5,5 | down | HS3ST1 | 9957 |
| 6,5 | down | CHRDL1 | 91851 |
| 3,4 | down | PROM1 | 8842 |
| 3,2 | down | HGF | 3082 |
| 3,1 | down | C1orf51 | 148523 |
| 3,6 | down | BBS12 | 166379 |
| 3,4 | up | THC2525418 |  |
| 3,6 | down | C14orf108 | 55745 |
| 4,1 | down | ZNF570 | 148268 |
| 3,2 | up | A_24_P928705 |  |
| 3,3 | up | DNPEP | 23549 |
| 4,4 | down | EGR1 | 1958 |
| 3,9 | down | D2HGDH | 728294 |
| 4,3 | up | DST | 667 |
| 3,2 | up | KCNIP2 | 30819 |
| 3,2 | up | ODC1 | 4953 |
| 3,7 | up | INHBA | 3624 |
| 3,1 | up | TCEB3C | 162699 |
| 3,5 | up | MDH2 | 4191 |
| 3,3 | down | DKFZP434O047 | 26083 |
| 3,3 | up | TAOK1 | 57551 |
| 3,0 | up | DIRC2 | 84925 |
| 4,5 | down | RKHD3 | 84206 |
| 3,3 | down | EGFL9 | 65989 |
| 4,1 | up | FANCM | 57697 |
| 3,2 | down | CEACAM1 | 634 |
| 5,0 | down | DSCR1L1 | 10231 |
| 3,2 | up | ROR2 | 4920 |
| 3,1 | up | KCNK13 | 56659 |
| 3,8 | up | RCCD1 | 91433 |
| 3,8 | down | RPS6KA3 | 6197 |
| 4,6 | down | SYT15 | 83849 |
| 3,4 | down | HEL308 | 113510 |
| 3,1 | up | PIAS3 | 10401 |
| 3,1 | up | GNPDA1 | 10007 |
| 4,6 | down | DOCK8 | 81704 |
| 7,5 | down | CHMP4B | 128866 |
| 3,2 | down | RRAS2 | 22800 |
| 11,6 | down | DMPK | 1760 |
| 9,4 | up | AA043564 |  |
| 3,1 | up | ZXDA | 7789 |
| 3,2 | up | FASLG | 356 |
| 4,1 | down | CCDC80 | 151887 |
| 4,8 | down | THC2668815 |  |
| 3,1 | down | KIAA1086 | 23217 |
| 4,0 | down | ENST00000390369 |  |
| 4,1 | up | AK001903 |  |
| 3,0 | down | RND1 | 27289 |
| 3,5 | down | PPP1R1A | 5502 |
| 7,5 | down | AU184995 |  |
| 3,1 | down | CHST11 | 50515 |
| 3,3 | up | CXCL12 | 6387 |
| 3,8 | down | TNC | 3371 |
| 4,2 | down | MYST3 | 7994 |
| 3,3 | up | RAXL1 | 84839 |
| 4,6 | up | CDH12 | 1010 |
| 3,6 | down | Z21967 |  |
| 3,3 | down | THC2681718 |  |
| 3,8 | down | MAP3K10 | 4294 |
| 3,6 | up | LOC728012 | 728012 |
| 3,7 | up | LOC283129 | 283129 |
| 3,5 | down | AK125162 |  |
| 3,1 | down | KRT72 | 140807 |
| 3,8 | down | PARD3B | 117583 |
| 3,9 | up | TMEFF2 | 23671 |
| 3,0 | down | ENST00000325863 |  |
| 3,3 | down | LTB | 4050 |
| 4,3 | down | FUS | 2521 |
| 3,3 | down | PRODH | 5625 |
| 5,2 | down | MSI2 | 124540 |
| 4,4 | down | CDC42BPA | 8476 |
| 3,4 | up | TFAM | 7019 |
| 5,3 | up | TMEM182 | 130827 |
| 5,1 | down | THC2618720 |  |
| 3,1 | down | EYA4 | 2070 |
| 3,9 | up | UCHL1 | 7345 |
| 3,9 | up | PCDHB6 | 56130 |
| 3,4 | up | A_24_P83928 |  |
| 3,1 | down | F2 | 2147 |
| 4,3 | down | AK123297 |  |
| 4,8 | down | EPC1 | 80314 |
| 3,5 | down | UNQ1887 | 121665 |
| 3,8 | down | MATN2 | 4147 |
| 4,5 | down | STK32C | 282974 |
| 3,1 | down | BQ926066 |  |
| 4,4 | down | MMP13 | 4322 |
| 4,1 | down | DDIT4 | 54541 |
| 3,1 | down | ATP1A4 | 480 |
| 3,2 | down | DAG1 | 1605 |
| 4,3 | down | ID3 | 3399 |
| 4,1 | up | TMEM108 | 66000 |
| 3,3 | down | CLMN | 79789 |
| 3,3 | up | LOC414300 | 414300 |
| 3,4 | down | A_24_P852099 |  |
| 5,1 | down | SEPP1 | 6414 |
| 3,3 | down | FLJ37798 | 401264 |
| 4,1 | up | A_24_P524462 |  |
| 3,3 | down | SLC24A3 | 57419 |
| 3,5 | down | SPOCK3 | 50859 |
| 3,1 | down | MYLK | 4638 |
| 3,3 | up | DDEF1IT1 | 29065 |
| 4,1 | up | NR4A1 | 3164 |
| 4,1 | up | A_32_P118959 |  |
| 3,7 | down | SSNA1 | 8636 |
| 3,0 | Down | FADS1 | 3992 |
| 4,7 | down | TACR1 | 6869 |
| 3,4 | down | CYBB | 1536 |
| 3,1 | up | A_24_P375132 |  |
| 4,2 | down | CEACAM21 | 90273 |
| 4,0 | down | SNTB1 | 6641 |
| 3,6 | down | MPP3 | 4356 |
| 3,2 | up | HCG3 | 414061 |
| 3,9 | up | SLC26A7 | 115111 |
| 4,6 | down | BMP2 | 650 |
| 3,6 | down | STK40 | 83931 |
| 4,5 | down | PMFBP1 | 83449 |
| 3,6 | down | BC024651 |  |
| 3,7 | down | THC2578835 |  |
| 3,1 | down | DNMT3A | 1788 |
| 3,7 | up | AF088076 |  |
| 3,3 | down | LPHN1 | 22859 |
| 3,2 | down | HS2ST1 | 9653 |
| 3,2 | down | FLJ45187 | 387640 |
| 3,5 | down | APH1A | 51107 |
| 3,9 | down | RKHD3 | 84206 |
| 3,1 | down | RFX3 | 5991 |
| 4,4 | down | PSAP | 5660 |
| 3,5 | down | TMEM42 | 131616 |
| 3,2 | down | LFNG | 3955 |
| 3,1 | up | ANKRD11 | 29123 |
| 3,4 | up | CD36 | 948 |
| 3,6 | down | ENST00000358431 |  |
| 3,5 | up | PBX1 | 5087 |
| 3,2 | up | NPAT | 4863 |
| 3,7 | up | KIAA0802 | 23255 |
| 4,1 | down | ID1 | 3397 |
| 3,4 | down | X03757 |  |
| 4,6 | up | CCDC102B | 79839 |
| 3,2 | up | THC2519126 |  |
| 4,1 | down | PPME1 | 51400 |
| 4,3 | up | THC2657193 |  |
| 3,5 | down | VDAC1 | 7416 |
| 3,5 | up | MITF | 4286 |
| 4,9 | down | BCL11A | 53335 |
| 4,2 | up | A_24_P931598 |  |
| 4,1 | down | VDAC1 | 7416 |
| 3,2 | up | MTHFD2L | 441024 |
| 3,1 | down | MOBKL2C | 148932 |
| 3,1 | down | PPP1R3F | 89801 |
| 4,5 | down | SETD5 | 55209 |
| 6,1 | up | MGC34824 | 285154 |
| 3,2 | down | TAF15 | 8148 |
